# From Mental Health Industry to Humane Care. Suggestions for an Alternative Systemic Approach to Distress

**DOI:** 10.3390/ijerph18126625

**Published:** 2021-06-20

**Authors:** Radosław Stupak, Bartłomiej Dobroczyński

**Affiliations:** 1 Institute of Philosophy, Faculty of Philosophy, Jagiellonian University, 52 Grodzka St., PL 31044 Kraków, Poland; 2 Institute of Psychology, Faculty of Philosophy, Jagiellonian University, 6 Ingardena St., PL 30060 Kraków, Poland; bartlomiej.dobroczynski@uj.edu.pl

**Keywords:** open dialogue approach, soteria, power threat meaning framework, drug-centered approach, mental health reform, critical psychiatry

## Abstract

The article proposes a rough outline of an alternative systemic approach to mental health issues and of a more humane mental health care system. It suggests focusing on understanding mental distress as stemming from problems in living, using medications as agents facilitating psychotherapy, or as a last resort and short-term help, according to the principles of harm reduction. It argues that understanding drugs as psychoactive substances and studying the subjective effects they produce could lead to better utilization of medications and improvements in terms of conceptualizing and assessing treatment effects. Qualitative research could be particularly useful in that regard. It also advocates a radical departure from current diagnostic systems and proposes a synthesis of already existing alternatives to be used for both research and clinical purposes. Accordingly, a general idea for an alternative mental health care system, based on a combination of Open Dialogue Approach, Soteria houses, individual and group psychotherapy, cautious prescribing, services helping with drug discontinuation, peer-led services and social support is presented. The proposition could be seen as a first step towards developing a systemic alternative that could replace the currently dominating approach instead of focusing on implementing partial solutions that can be co-opted by the current one.

## 1. Introduction

According to the World Health Organization, depression is the leading cause of ill health and disability worldwide, with more than 300 million people considered to be living with the diagnosis, an increase of more than 18% between 2005 and 2015 [1]. Whitaker [2] paints a compelling picture of our current treatment and research paradigms actually making things worse, and his position is partly or fully supported by many other researchers, e.g., [3,4,5]. Former National Institute of Mental Health (NIMH) president, Thomas Insel, bitterly summarized his 13 years of NIMH presidency admitting that $20 billion spent on biological research failed to “move the needle in reducing suicide, reducing hospitalizations, improving recovery for the tens of millions of people who have mental illness” [6]. In the United States, the 65% increase in antidepressant use between 1999 and 2014 [7] has been accompanied by the age-adjusted suicide rate increase of 33% from 1999 through 2017 [8]. The biopsychosocial model (which actually seems to be a “bio-bio-bio model,” according to the American Psychiatric Association (APA) former president [9], needs a radical overhaul. This model is characteristic mainly of Western or Global North countries; however, there are ongoing efforts and calls to export it globally [10]. Biologically oriented psychiatric research may eventually, despite the costly failure to date, produce valid biomarkers or mechanistic explanations, but so far, even if it succeeded in expanding our knowledge in neuroscience, it has failed to address the primary goal psychiatric research should serve—helping patients. The progress in neuroscience does not seem to translate into better treatments, and new drugs are no better than those discovered by accident in the middle of the 20th century and work on the same underlying principles [11].

Even though it would be tempting to focus on criticizing the current model, we would like to instead turn our attention predominantly towards arguing for specific systemic solutions. Our aim here is not a systematic review of alternative approaches, but rather a conceptual analysis based on a narrative review in order to tentatively propose a foundation for a comprehensive framework based on selected mutually theoretically and practically compatible solutions. By creatively synthesizing already existing theoretical and practical approaches, we could envision a system that could replace the current one.

We will propose a few principles around which a more humane mental health care system could be organized and a rough outline of a possible solution. This system could be based on a combination of the Open Dialogue Approach, Soteria houses, individual and group psychotherapy, cautious prescribing, services helping with drug discontinuation, peer-led services and social support. We will focus on mood and psychotic disorders while discussing it, but there is no reason to think that such a system could not be useful in regards to other diagnostic categories. We will also propose some solutions for research practice regarding research methodology, important research questions and, last but not least, diagnostic systems. We will argue for understanding mental distress as stemming from problems in living and transforming the research agenda accordingly by utilizing the Power Threat Meaning Framework [12,13] and focusing on specific complaints [14]. Accordingly, the subjective effects of drugs and outcomes of other interventions could be studied in relation to these frameworks with wider utilization of qualitative research methods in order to focus on the perspective of service users.

We will be using some of the standard nomenclature for stylistic reasons and in order to avoid more confusion, but it has to be noted that, for example, speaking of “symptoms,” while the existence or presence of any underlying disorder is doubtful, may be misleading, or that words like “intervention” convey a specific kind of relation (especially in terms of power), which in fact may be harmful to “patients.” Similar problems arise with regards to words such as “disease,” “illness” or “disorder” [15,16] and many others. The role of language will also be very shortly addressed in appropriate sections later in the text.

## 2. The State of Affairs

### 2.1. Clinical Practice

There is a substantial diversity of settings in terms of mental health care across countries and within countries, even within Global North, high income or European Union countries. The Lancet Commission on Global Mental Health and Sustainable Development acknowledges this while still providing recommendations to be implemented globally [17]. A systematic comparison of different systems across the world with all their nuances and subtle differences would itself require a monumental work; however, it seems that it would not be an overstatement to claim that, globally, the biomedical model of mental health is dominating, even if there seem to be emerging trends for the inclusion of a more nuanced perspective and a bigger emphasis on psychosocial interventions. The interventions are still often based on a predominantly biomedical perspective and could be seen as an extension of the biomedical model, where the “psychosocial” has been, in fact, colonized by the “biomedical” [18].

Several examples from different countries may help in substantiating this claim. Even though it may seem to be a controversial and contested statement, depression could be considered to be a disorder that can be treated both pharmacologically or psychotherapeutically with similar effectiveness, regardless of severity or symptom profile, as indicated by a recent meta-analysis [19]. Thus, it may be worthwhile to focus on data concerning antidepressant prescription, psychotherapy and depression in order to portray systemic clinical practice.

In the United Kingdom, which has one of the highest levels of public spending on mental health in Europe, a report from the Mental Health Taskforce to the NHS stated that the provision of psychological therapies for people with anxiety and depression has expanded hugely in recent years, which meant that 15% of needs were met and recommended reaching a goal of 25% by the year 2020–2021 [20], meaning an increase from 900,000 to 1.5 million people [21]. It was reported that in 2019–2020, there were 1.69 million people referred to psychotherapy, and 1.17 million people started psychological treatment (with 6.9 treatment sessions on average) [22]. By comparison, the number of people receiving prescriptions for antidepressants in the UK was growing steadily at about 200,000 a year during the last 5 years, rising from 6.8 million in 2015–2016 to 7.8 million in 2019–2020. There was, however, a decline in the people prescribed anxiolytics and hypnotics from 2.4 million to 2.1 million [23]. These numbers seem to indicate a huge disproportion in the utilization of psychological and biological therapies.

Similarly, in Poland, with one of the lowest levels of public expenditures on mental health in Europe, public spending on psychotherapy and psychological interventions for depression amounted to just over 20 million PLN, while expenditures on psychiatric consultations (40 million) and pharmacotherapy (130 million) for depression totaled over 170 million PLN (and more than 300 million PLN of not subsidized spending on antidepressants). The difference between public funding for psychotherapy and pharmacotherapy of depression suggests a bias in favor of biomedical interventions. The defined daily dose of subsidized antidepressants rose by about 60% between 2013 and 2018, and the number of people receiving prescriptions for these drugs went up by almost 300,000 from 948,000 to 1.3 million. It may be worth noting that in the official report documenting public expenditures on the treatment of depression, “psychotherapy” is mentioned 4 times, “pharmacotherapy” 14 times and “medications” 99 times, while most of the report deals with assessing spending on particular drugs and medication adherence [24].

Still, it is estimated that in the USA, medication alone was used in just 11.2% of cases, psychotherapy alone in 47.3% and a combination of psychotherapy and pharmacotherapy in 36.2% (with 5.3% receiving no treatment). However, psychotherapy visit frequency had a median of two visits per month in the first month following the diagnosis and dropped to zero by month 3, which may be associated with insurance coverage, especially as patients in a high-deductible health plan had the highest utilization of medication-only treatment [25], and means that most patients receive a very small number of psychotherapy sessions. This can be contrasted to antidepressant use, with 68% of patients using antidepressants for 2 years or more, 1 in 4 patients for 10 years or more and 12.7% of people older than 12 reporting antidepressant use in the past month (2011–2014 data) [7]. Another study reports that the proportion of patients who were treated for depression using psychotherapy has decreased from 53.7% in 1998 to 50.4% in 2015, while the proportion of those receiving pharmacotherapy remained steady at around 80% [26].

In 2018–2019 in Australia, regardless of diagnosis, 4.3 million people received mental health prescriptions, of which 70.9% were antidepressant medications, while only 453,000 patients received community mental health care services, an almost 10-fold disproportion [27]. Australia has one of the highest per-capita consumers of antidepressants among OECD countries, and there was an almost 100% increase between 2002 and 2018 [28].

The prescribing of antidepressant medications has also increased substantially in China. In a small sample of studied hospitals, a 42.6% increase between 2013 and 2018 has been reported [29]. Overall, in OECD countries, antidepressant consumption doubled between 2000 and 2015, while almost tripling in some countries and an almost five-fold growth noted in Slovakia [30]. There seems to be an increase in long-term use of antidepressants which may partly explain a growing trend in overall antidepressant consumption [31].

It was estimated that the global antidepressant market would double revenues in 2020 due to the COVID-19 pandemic, rising from 14 to 28 billion USD [32], which may also suggest that the dominant response to the mental health aspect of the crisis relies primarily on medications, even as there were concerns raised about the safety of these drugs in relation to the virus [33]. Data on the number of people accessing psychosocial services in different countries is much harder to find or even non-existent and may also reflect a bias in favor of pharmacotherapy. Regardless of the countries income group, there are substantially more psychiatrists and other medical doctors working in the mental health sector than psychologists, social workers and occupational therapists combined [34], which also seems to indicate a global dominance of a biomedical approach, even if there are some countries that are an exception to this rule. An extreme example of this domination may be the fact that homeless people in New Zealand are offered antidepressants and antipsychotics more often than any other kind of help [35].

### 2.2. Research Practice

As for research practice, there are some studies trying to assess expenditures and policies; however, they also target narrow research areas or specific countries or simply include mental health as a part of biomedical research. The RAND Corporation report [36] is probably the only one examining the global funding landscape for the whole of the mental health field. It also employed a bottom-up approach (analyzing research output and funding acknowledgments) instead of a top-down analysis (looking at funding agencies), which allowed for identifying funders omitted in many other studies.

According to the report [36], Global North countries dominate the global research landscape. Mental health research funders are located primarily in North America, Northern and Western Europe and China, with the United States as both the largest producer of research and the biggest source of funding. The top 4 most often acknowledged funders stem from the USA (US National Institutes of Health, NIMH, National Institute on Drug Abuse, National Institute on Aging). Top 30 funders represent the following countries: USA, Canada, Australia, European Union (as a distinct entity from the particular countries of the EU), China, UK, Netherlands, Germany, Spain, Brazil, Japan, Sweden, and together they are responsible for the funding of 96% of published research, with the USA alone having a 56% share. This may reflect the role of the United States agencies, and indirectly that of APA and American Psychological Association, in shaping the global mental health discourse and practice.

It is difficult to assess what kind of research precisely gets funded and published. The RAND report distinguishes four levels of research while providing journal names that are supposed to reflect the type of research published; these are: 1. clinical observation (e.g., *Schizophrenia Bulletin*), 2. clinical mix (e.g., *Journal of Psychiatric Research*), 3. clinical investigation (e.g., Neuropsychopharmacology) and 4. basic biomedical (e.g., Neuroscience). Estimating what percentage, if any, represents studies dealing with psychosocial interventions and factors within these categories would require a dedicated research project; however, even the category names, journals provided as examples and their research scope may suggest that it is a rather modest share. For example, even though *Schizophrenia Bulletin* editorial policy states “[w]e view the field as broad and deep, and will publish new knowledge ranging from the molecular basis to social and cultural factors.” This is immediately followed by: “[w]e will give new emphasis to translational reports which simultaneously highlight basic neurobiological mechanisms and clinical manifestations,” and there is no mention of psychology or psychotherapy [37]. The *Journal of Psychiatric Research* mentions that it “is dedicated to innovative and timely studies of four important areas of research” and includes “environmental, social, psychological and epidemiological factors” along with “biochemical, physiological, genetic” in one of them, while the remaining three seem strictly biomedical [38]. One of the biggest funders of mental health research globally—NIMH—has been criticized for completely abandoning psychotherapy research in favor of a strictly neurobiological approach [39]. Still, even if the number of psychosocially oriented studies is relatively small, a noticeable minority exists, and this paper is in large part based on it.

A recent meta-analysis of interventions for suicide and self-injury may perhaps serve as a more convincing example of the situation [40]. The study identified 1125 unique RCTs, 816 dealt with medication only, 80 with psychotherapy and medication combined, and the remaining 229 concerned all other kinds of psychosocial interventions (e.g., 5 RCTs of psychoanalysis). It may be worth noting that medications had minimal effects, confidence intervals of other interventions were relatively wide, possibly due to small sample sizes, and there was no improvement in terms of outcomes during the last 50 years.

### 2.3. Historical and Conceptual Context

Two events seem pivotal to the genesis of this situation and the dominance of the biomedical approach, namely: the accidental discovery of the psychotropic effect of chlorpromazine in 1952 and the publication of the third edition of the *Diagnostic and Statistical Manual of Mental Disorders* (DSM-III) in 1980 [41].

The DSM-III and its subsequent editions were a return to a 19th-century tradition of Emil Kraepelin. In a panegyrical article, Compton and Guze [42] compare it to the approach of Linnaean botanists, where careful observation and description lead to the creation of categories. In this approach, equated with the medical or disease model, symptoms lose all of their underlying meaning, and they have an external validity of their own. A mere presence of a symptom is what is crucial, its subjective meaning or other qualities—irrelevant. The switch from mainly psychoanalytic conceptualizations to an allegedly objective descriptive language was meant to counter some of the problems highlighted by the antipsychiatry movement of the 60 s and 70 s and, at the same time, provide psychiatry with the authority of hard science [41]. Robert Spitzer, DSM-III chair, cynically concluded that it “looks very scientific. If you open it up, it looks like they must know something” [43]. Allen Frances, DSM-IV chair, admitted that “we knew that everything that came before was arbitrary,” so “it felt better to stabilise the existing arbitrary decisions than to create a whole assortment of new ones” [44].

In 1975, Arnold Ludwig, in a commentary symptomatically titled *The Psychiatrist as Physician* [45], put forward the basic assumptions behind this change, namely that “a sufficient deviation from normal represents disease, that the disease is due to known or unknown natural causes, and that elimination of these causes will result in cure or improvement in individual patients” (p. 603), “the mental disease is assumed to arise largely from "natural" rather than metapsychological, interpersonal, or societal causes” (p. 603). Interestingly, in this approach disorders “produced primarily by psychosocial variables” (p. 604) were to be excluded as affecting people with “intact neurophysiological functioning” (p. 604). Still, the definition of “normal” represents a serious philosophical problem and in practice seems to be more a case of value judgments than biological or observational data [46].

The APA states on their webpage that: “[m]ost medications are used by psychiatrists in much the same way that medications are used to treat high blood pressure or diabetes” [47]. Even if we consider APA to only represent American psychiatry, it is fair to say that it sets the tone for global psychiatry, not only through the DSM. The statement can also be considered to be only a simplified message for the lay people that most psychiatrists would not endorse, but we would then have to come to the conclusion that APA is consciously misinforming the public. Rather, it seems that it is a statement reflecting the way most practicing psychiatrists actually think about psychiatric illnesses and psychiatric medications.

The dominance of this biomedical model is acknowledged in a recent report of UN Special Rapporteur, stating that: “[m]ental health systems worldwide are dominated by a reductionist biomedical model that uses medicalization to justify coercion as a systemic practice and qualifies the diverse human responses to harmful underlying and social determinants (such as inequalities, discrimination and violence) as “disorders” that need treatment” [48] even though there are serious doubts about the utility of an approach based on unreliable diagnoses of unknown validity [41]. Precision psychiatry heralded as a solution to some of the problems of the biomedical model seems to be based on the same flawed assumptions [49].

## 3. Alternative Theory and Research

### 3.1. Etiology—Problems in Living

The first thing that needs addressing is the question of the etiology of mental disorders (if we, for a moment, agree to call specific configurations of manifestations of suffering “disorders”), as this understanding projects onto all other domains. There is now a wealth of research highlighting the role of social, economic and other factors, such as adverse life events, on the probability of being diagnosed with a mental health problem, even psychosis. They have been shown to be causally associated [50]. Some of them have much higher relevance than any known biological or genetic correlates [51]. To mention just a few examples, financial and social status [52,53], belonging to an ethnic minority [54], or being a victim of child abuse and other adversities [55] have been shown to influence the frequency of particular diagnoses and prescription of psychiatric drugs. A longitudinal study revealed that financial problems of British students precede depression and alcohol abuse [56], and difficulty paying back debts precede common mental disorders in the adult population in the Netherlands [57], pointing to economic factors as causal. This clearly shows that cultural and social determinants must not be ignored or even that they should be given priority, and psychiatric disorders could be seen as consequences of frustrated human needs [58]. As Longden [59] puts it: “an important question in psychiatry shouldn’t be what’s wrong with you but rather what’s happened to you.”

Still, the prevailing narrative is one of the biological mechanisms or abnormalities that should be corrected with drugs (as biological illnesses need biological treatments), and this narrative, among many other issues, shapes the way mental health care systems and mental health awareness campaigns are designed and financed. One particularly worrying consequence of this, in the context of individual suffering, is the fact that some people with psychiatric diagnoses may even lose the ability to understand their mental states as something that is directly connected to the lives they live, e.g., the suffering of somebody who was abandoned in a romantic relationship could be explained by themselves (and mental health professionals) as a “relapse of depression”—one caused by “chemical imbalances”—without realizing at all and disregarding the impact of the psychological or social situation [60]. The use of medication can also have a detrimental effect on identity [61].

Understanding psychiatric disorders as primarily consequences of various life circumstances and their meanings for individuals would require a radical reshaping of mental health care, but this could lead to improvements in terms of outcomes (to use medical language) or quality of life and life satisfaction in general (to use a more neutral one) for those suffering and societies as a whole. Improvements that biological psychiatry has largely failed to deliver, as current treatment outcomes for depression and schizophrenia are probably worse, especially long-term, than in the first half of the 20th century, before the era of pharmacotherapy [62,63]. It could also be argued that if the mental distress people experience is seen as problems in living instead of specific disorders, speaking of etiology or pathomechanisms becomes self-contradictory. However, individual psychological factors definitely play a role, which may explain why some people are affected more by adverse circumstances, and so it is not necessary to posit inherited (or inherent) biological differences to account for this. Even if they exist, they are not necessarily the primary cause, as they may well be a consequence of psychological and social situations—after all, brains are shaped by and adapt to the environment. Appropriate actions would have to follow this kind of understanding of mental distress, and perhaps the most important thing that should be addressed and changed is the way drugs are used and their action conceptualized in mainstream psychiatry.

### 3.2. Understanding and Using Medications

The prevailing narrative concerning psychiatric drugs is that they are “magic bullets” targeting disorder-specific biological abnormalities. This discourse is so strong that a refusal to use psychiatric drugs is often equaled to a lack of insight, even when a patient seems quite rational in all other aspects, while consent for drug use is equaled with better insight, even when patients express otherwise bizarre claims about themselves and their condition [64]. The dominating approach is what Moncrieff [5] calls a Disease-Centered Model of Drug Action, while advocating instead for a Drug-Centered Model of Action. In the latter, psychiatric medications are understood not as agents that correct some kind of pathology or cures for disorders but rather as something that actually creates abnormal bodily states. Their potential usefulness in alleviating suffering comes precisely from this quality, which allows them to suppress, blunt, alter or enhance subjective experiences. This means that psychiatric medications could be understood essentially as psychoactive agents, not that different from alcohol or illicit drugs and with similar potentially harmful consequences regarding both biological and psychological levels. If we look at psychiatric disorders as primarily meaningful reactions to life events, our goal is then not to cure a disease but to help in overcoming life difficulties. Psychoactive effects of drugs may be helpful in that.

Conceptualizing medications this way has many consequences for the way they should be used in clinical practice and how their action should be studied. Research could rely more on qualitative methods in order to understand what is really the subjective experience of taking psychiatric drugs (and what is really the experience labeled as a disorder requiring drugs). Currently used instruments focus on predefined outcome measures related to the symptoms that diagnostic categories consist of, and these may not really correspond to the actual psychoactive effects the drugs exert. Moreover, a predefined set of criteria/symptoms streamlines both the researcher and the patient into a presupposed mode of understanding and forces distortion and deformation of actual phenomena in order to fit them into ready-made check-boxes. If there is no box for something, it gets overlooked, as if it was not there.

Thinking of drugs as acting on symptoms can be misleading also because of other factors, e.g., in the case of antihistamine drugs, the same effect—sedation—can be regarded as a side-effect (in the case of treatment of allergies) or as a desired therapeutic effect (in the case of reducing the expression of symptoms of psychosis). For drugs used in psychiatry, the important question, in this case, should be how much the “symptom reduction” in fact depends on the psychoactive effect of sedation, how does the sedation make the patient feel and if the effect is perceived as beneficial and desirable or if the opposite is the case: as harmful and unwanted, especially with regards to social and interpersonal functioning. The subjective psychoactive effect of a drug should be at the center of our attention. The same applies to “energetic,” “numbing-down” or other properties of antidepressants and different classes of psychiatric drugs. In general, the focus should move away from research about symptoms towards questions about how the drugs actually make people feel.

These differences in perspectives are well pictured in the following quotes of psychiatric survivors:

“I did not manifest any of my internal distress, because I did not show any evidence of internal life at all. This is not the same as the absence of madness. Yet it was the gauge by which the success of treatment was measured.”

[65]

“I found the medication made me feel empty and soulless, I could not think past considering my basic needs. The psychiatric drugs made me physically weaker and affected my hormones so I became during this time impotent. I was concerned about this. However, to the outside world because of the mind-numbing effects of the drugs I was less focused on my spy and spiritual beliefs. The doctors pronounced that I was responding well to the medication.”

[66]

Standard clinical instruments would probably show an improvement, perhaps even a remission, while, obviously, the patient, the subject, could, in fact, be even more miserable. The same applies to the study of treatment outcomes in general, often failing to include patients’ perspectives on what constitutes an improvement and using imposed and often prejudiced criteria of what counts as a therapeutic success [67]. A striking example of this fact is that in the Hamilton Depression Rating Scale, attributing depression to social causes is understood as poor insight and worsening of depression [68]. It seems that in other branches of medicine, patients’ opinions are given more importance than in psychiatry in terms of assessing patients’ condition and treatment outcomes, even though psychiatry lacks objective measures (e.g., glucose levels, X-ray pictures, etc.) that are available elsewhere. Paradoxically, while the dominant model in psychiatry deals mainly with the brain and private phenomena, it relies almost exclusively on a subjective third-person perspective (of physicians, family members, society in general) for assessment, especially in short-term clinical trials.

However, from the utility point of view of clinicians and patients, particular nerological and biological mechanisms are secondary, even though they may be also important, especially for understanding the effects of long-term exposure to drugs and withdrawal [69]. In practice, clinicians often try to match “symptoms” with the supposed psychoactive effect of drugs, anyway, by relying on their pharmacological profile and assumed relation between the mechanism of action and subjective effects they may produce [70]. This kind of research agenda would, then, fit the actual practice better and allow for better utilization of drugs.

That said, the use of medications should be completely reconceptualized, especially given that, as mentioned earlier, it is possible that the current paradigm does more harm than good. Understanding drugs according to the Drug-Centered Model of Action would suggest using medications as a last resort for long-term therapy, only when other options have been tried and failed or when the patient is convinced that this is the best solution available. This would require a truly informed consent on the side of the patient informed about possible risks and harms and still a careful and responsible assessment of the prescriber, especially in the case of drugs such as benzodiazepines, which are now well established to be habit-forming, or the emerging data about common, long-lasting and severe SSRI withdrawal symptoms [71,72].

#### 3.2.1. Drugs as Short-Term Help

Certainly, in some circumstances, pharmacologically induced sleep, for example, is better than no sleep at all, but that does not necessarily mean that prolonged use of hypnotics, sedatives or neuroleptics is indispensable or beneficial. Drugs could be then primarily used as short-term solutions helping to overcome specific temporary difficulties, in a somewhat similar way as one can drink a cup of coffee to fight fatigue or have an alcoholic drink to relax. Research on the psychoactive properties of psychiatric drugs could provide important information on when and how these drugs could be used this way. An important fact to consider is that promoting drugs as correcting biological abnormalities may promote life-long use and hinder discontinuation of treatment even when it is no longer necessary [73] while thinking of medications as psychoactive substances could have the opposite effect and promote shorter use. Regardless of the way drugs are actually used and studied, more research addressing the question of the methods of safe discontinuation of psychiatric medications is urgently needed, as withdrawing from psychiatric medication is poorly understood and confounds the results of maintenance trials [74]. This, as well as the study of iatrogenic effects of psychiatric drugs, may be the area of biological studies that would be particularly useful.

#### 3.2.2. Drugs as Facilitators of Psychotherapy

Another way of looking at psychiatric drugs would be to think of them as substances that may facilitate psychotherapy and/or as agents inducing lasting changes thanks to the subjective psychological states they produce. Examples of such an approach would be the current studies on the use of 3,4-methylenedioxymethamphetamine (MDMA) for the treatment of Posttraumatic Stress Disorder, where the psychoactive effects of MDMA are thought to allow for a specific therapeutic relationship to be established [75,76]. Other examples would be the use of psilocybin [77], LSD [78], ketamine [79,80] or ayahuasca and other substances for various disorders. It is important that the effects of such interventions are not reduced to the neurobiological level only. This seems to be already the case with the, patented in the form of a nasal spray, enantiomer of ketamine, which was recently approved for the treatment of treatment-resistant depression, despite poor evidence of effectiveness [81]. As one of the authors of a recent trial [82] comparing psilocybin and escitalopram commented: “if psilocybin becomes just another drug, it will be as uninspiring and ultimately disappointing as SSRIs have been for many” [83].

The relation between the neurobiological and psychological or subjective is another complicated philosophical problem, but the insistence on interpreting the action of psychedelic substances on the physiological level, which leads to the goal of avoiding altered states of consciousness in therapy, could explain the poor results of ketamine nasal spray. In fact, it is precisely these states that may carry the therapeutic potential or effect [84,85]. It is important that in the process of implementation of this kind of therapies the traditions from which some of these substances and practices were appropriated are not erased and indigenous knowledges treated as equal contributors, especially as they can provide a much wider social and cultural context for the interpretation and the consequences of the experience [86]. However, it may also seem that the particular psychological and cultural frameworks of interpretation are secondary, i.e., similar insights could be successfully articulated within Western discourse.

Generally, our thinking about using psychiatric drugs could follow the principles of harm reduction, as in the case of illicit drug use, not only in the cases of withdrawing [87] but as a guiding principle. Drugs should be used voluntarily (as all other services—otherwise speaking of “service users” is merely a linguistic distraction; someone who is treated against their will is neither a “user” nor a “consumer” [88]) and rely on an actual informed consent.

### 3.3. Diagnosis

Kinderman [89], a former President of the British Psychological Society, goes as far as describing the current diagnostic systems as “invalid and inhumane and even bizarre,” and he is not alone in this opinion. Critique of current diagnostic systems from many different perspectives and backgrounds is abundant [90,91]. A recent study concludes that DSM-5 diagnoses are so heterogenous it makes them practically useless [92]. It is important to remember that the same complaint (e.g., anxiety) can be associated with different causes, as well as that the same cause (e.g., death of a loved one) can lead to completely different complaints or involve different psychological mechanisms. This holds also on the biological level and the causal relation between different brain states and subjective phenomena—similar experiences may be caused by different brain states and vice-versa.

What is crucial is that diagnoses could be considered to be the primary source of stigma, self-stigma, power imbalances within the psychiatric system and an excuse for forced treatment and violations of human rights that effectively produce second-class citizens [93,94]. Some studies suggest that using another terminology, e.g., psychosis instead of schizophrenia, could be beneficial and reduce stigma and discrimination [95]. However, it is unlikely that such an effect would be longstanding. It is reasonable to assume that as soon as the new label is understood to refer to a similar construct, discriminatory beliefs will be added also to the new word. Language in psychiatry is important [96], yet changing the terms alone will not help if the meanings and practices associated with them stay the same. As one patient said: “(...) since I got sick I still think that it shows and that I have to be careful not to give myself away” [97]. It is possible that this stress, even according to the stress-vulnerability model, can lead to worse outcomes playing the same role minority stress does for the LGBTQ population [98]. Framing mental distress not as a deficit or a “malfunction” but as primarily a sign of human potential and opportunity to grow, as in Kazimierz Dąbrowski’s theory of positive disintegration, could mitigate this stigmatizing and self-stigmatizing effect [94].

Bearing in mind all the negative consequences associated with the reliance on diagnostic classifications and the explicit and implicit meanings behind them [99], it might be best to replace them with another approach. Diagnoses are already neither necessary nor sufficient for the provision of services [100], so even the common response that they are indispensable because of administrative reasons or the interests of insurance companies does not seem justified. Services could be provided in a model more resembling social care, where labeling is not necessary. For clinical and research purposes, focus on specific complaints [14], and the Power Threat Meaning Framework could be utilized [12]. That is, we should be asking questions such as: what has happened to you? how did it affect you? what sense did you make of it? what did you have to do to survive? instead of going over symptoms checklists to arrive at a diagnosis. This would go in tandem with the focus on the psychoactive properties of drugs sketched earlier, as specific problems could be matched with specific psychoactive properties. It could also help the patients to conceive themselves as active and responsible agents, which would have an empowering effect, in contrast with being a passive victim of an externalized, yet internal and biological abnormality.

## 4. Alternative System—Clinical Practice

Practical clinical solutions that would fit the propositions described above and allow for the development of an appropriate mental health system already exist. Such a system could be founded on several basic elements: 1. The Open Dialogue approach serving as the basis and first contact or referral point; 2. Soteria houses for people needing stationary care; 3. individual and group psychotherapy of different modalities in cases where Open Dialogue or Soteria are not necessary; 4. regular screening for adverse reactions for people using drugs and services allowing safe withdrawing; 5. self-help and peer-run services funded by the system as continuous support and rehabilitation; 6. individual social care regarding housing, everyday activities and supportive employment services.

### 4.1. Open Dialogue Approach

Firstly, the Open Dialogue Approach [101] developed in Western Lapland, Finland, could serve as the basis for such a system. It is important to remember the approach is not only a therapeutic modality but, originally, also a pragmatic and practical systemic solution to the institutional side of the organization of mental health services—it may be an important contribution to its success as the specific techniques and recommendations concerning therapy.

Open Dialogue could be described as a crisis intervention that takes the form of a unique narrative and systemic approach to family therapy delivered by mobile teams of professionals. Its main principles are: 1. the provision of immediate help—an initial network meeting convened within 24 h of first contact; 2. social network perspective—all key members of the social network are invited to the first meeting (including important people or officials who are not part of the family); 3. flexibility and mobility—no exact treatment plans are made during a crisis, methods are adapted to each case and change in response to current needs; 4. responsibility—the staff member who is first contacted is responsible for organizing the first meeting and the whole team is then responsible for the entire process; 5. psychological continuity—the same team is responsible for the treatment; 6. tolerance of uncertainty—for the first two weeks, frequent (daily) meetings are necessary to build a sense of security and avoid premature conclusions and decisions about treatment, especially regarding utilization of drugs; 7. dialogism—the goal is to foster a dialog that will increase patient’s sense of agency and allow development of a new understanding of the situation [102,103]. This approach corresponds to the framework regarding diagnosis presented earlier, and though it was developed as an intervention for psychotic disorders and is successfully used in these cases [104], it is safe to assume that it could be appropriate and beneficial in most other situations or diagnoses [105].

In this approach, diagnostic labels become secondary or even irrelevant as problems are seen as socially constructed narratives, not diseases. The central idea behind the principle of “dialogism” is the notion of “polyphony of voices,” both within subjects and between them. In this way, the focus is not on classifying and intervening but on fostering a dialogue in which all participants are treated equally in order to mobilize the resources of families and other social networks of the patient. The inclusion of different perspectives in order to allow for the development of healing, instead of pathologizing and disempowering, meanings of the situation is crucial. There is, however, no preplanned map for the stories or a predefined goal that the team is directing the system to achieve. Instead, the clinicians, understood as embodied emotional agents present in the moment, are focused on responding to clients empathetically while making sure that everyone is heard and does not feel abandoned, excluded or ignored, and communication is reestablished [106]. This approach is consistent with the research agenda and the assumptions of the Power Threat Meaning Framework [12] described above.

Even though on the surface it may seem that the principles of Open Dialogue are already widely present in mental health practice, at least in highly developed countries, a recent study describing experiences with the implementation of an approach based on the Open Dialogue in the USA has found the staff to report ”powerful changes in clinical practice, such as being more curious to listen, not having an agenda, realizing the answer to problems lie within the network, slowing things down and generating more dialogue” [107]. This way of working was perceived as weird by other mental health professionals, and this, as well as resistance to change of organizational culture, was reported as a challenge to implementing Open Dialogue. The participants also mentioned less burnout and better relationships among staff, clients and their families.

### 4.2. Soteria Houses

However, it may not always be possible to act accordingly with the Open Dialogue Approach, if, for example, the situation may be too difficult (it could be argued that, e.g., in rare cases of catatonia, a dialogue would be impossible), the system unwilling to cooperate (in order to, e.g., reestablish communication with somebody classified as having a “gross thought disorder or “incoherent speech”) or the provision of basic needs such as a safe place or food must take priority. In such cases, stationary care is necessary, and it could be based on the Soteria House model [108,109]. The Soteria approach shares some similarities with the Open Dialogue approach, both theoretically and practically: the metaphor of illness is rejected, problems are seen primarily as consequences of interpersonal or systemic relationships, the meanings attributed to them, and as developmental crises.

Essentially, Soteria is a home-like facility, with two members of the staff and up to eight patients, partly run by peers, with minimal hierarchy, where “being with” people in distress (instead of “doing to”—as in the Open Dialogue Approach, the staff primary responsibility could be described as facilitating a dialogue leading to a new understanding of the situation) and the importance of interpersonal relations are emphasized. Patients are responsible for the day-to-day functioning of the home, which provides a safe space that allows facing the crisis while preserving personal autonomy. The approach to drug use described earlier fits well with both Soteria and Open Dialogue models and is, in fact, close to what was practiced in the original Soteria [110] and is practiced today in Western Lapland [103]. Specifically, drugs are seen as agents that can provide temporary relief, not as curative agents, and are never used without a patient’s consent.

### 4.3. Individual and Group Psychotherapy

It may also be the case that intensive, system-wide intervention is not necessary, and the problem could be solved with fewer resources engaged or that the patient prefers another approach. Thus, the Open Dialogue team could decide to refer the patient to individual psychotherapy, and the meetings would provide the opportunity to jointly decide with the patient what kind of help or which therapeutic modality could be most beneficial. Trauma-Informed-Services could be particularly useful [111]; however, all approaches that allow for developing a personal meaning and ways of overcoming the problem, stemming from diverse theoretical backgrounds, could be helpful as long as the service user is free to choose and find the one that suits him or her [112]. This should enable a pluralistic approach to different problems and prevent a shift from one narrow range of options to another. It may seem that, in reality, the possibility to have a comfortable, caring, respectful conversation with another person is what is most important and helpful in a vast majority of cases [113,114].

### 4.4. Screening for Adverse Effects of Drugs, Services Helping with Discontinuation, Peer-Led Services and Social Support

In cases where a patient decides to use drugs long-term on a regular basis, the system should also offer regular screening for possible side and adverse effects and services to those people who later decide they would like to reduce doses or discontinue drug use. In developing and providing these, a close collaboration with service user initiatives is crucial [115].

Another important part of the system that could replace current rehabilitation schemes could be based on peer-led or peer-run services funded by the system. These could include self-help groups, such as Intervoice [116]. The goal of such interventions should move away from the elimination of “symptoms” towards learning to cope, attaining a better quality of life, developing an individual understanding of the situation and empowerment. It is important that peer-led initiatives stay as autonomous as it is possible, with minimal input from the professional administration regarding training, supervision, recruitment, etc., to avoid imposing an institutional view and character of these services [117]. Clubhouses [118] could form another part of this segment of the system. Organized help with ordinary duties of everyday living is also necessary, and it could be provided by social workers in a “social care” model. Provision of independent housing (with no live-in staff) and supportive employment services are also necessary [119], as are income support and occupational and financial assistance [120].

## 5. Human Rights

A system such as this one, together with the suggested approach to diagnosis and research, would have also been closer to the standards set by the Universal Declaration of Human Rights and the UN Convention on the Rights of People with Disabilities [121]. As psychiatric survivors point out, responding to a World Psychiatry issue that called for ignoring UN recommendations, the debate on this crucial human rights aspect of the mental health system often takes place without people directly affected by it [93]. Compulsory psychiatric treatment, coercion, direct use of force, restraints, community treatment orders and other discriminating actions and regulations seem to be a direct consequence of an inhumane system that many patients prefer to avoid, often because of the very coercion possibly involved, even if they would have voluntarily sought help otherwise [122]. Suicide prevention is a good example of the problems associated with compulsory treatment, and in this case, restricting freedom on the basis of risk assessment while acknowledging that we cannot reliably predict the risk [123] is, at best, paradoxical and seems counter-effective [124]. Another interesting paradox is that people deemed dangerous to themselves or others are often secluded with other such people as if the danger they supposedly present is acceptable as long as it is only the other inmates who are endangered. This is another aspect of stigmatization and dehumanization of people labeled mentally ill [125]. We feel that in a system in which people do not have to be afraid that they will be abused, involuntary treatment will not be necessary. The situations that lead to the use of force often stem from an inappropriate attitude of staff and serve discipline purposes only, and the use of restraints sometimes leads to death [126]. In general, we think that any form of involuntary treatment in mental health could only be justified in very specific circumstances, such as treatment of people convicted for serious crimes—for the duration of the sentence—and should be avoided at all cost or even made impossible.

## 6. Conclusions

As Burstow [127] rightly stressed, it is (or it should be) impossible to think about mental health outside of a wider—indeed planetary (economical, educational, political, environmental, etc.)—context. There will hardly be better mental health without a better world. Since socioeconomic factors and trauma or child abuse play a major role, it might be necessary to address them first not only screen for them. One such solution could be the introduction of Universal Basic Income [128]. Though calls to focus on “social interventions” start appearing even in “mainstream” psychiatry journals [129], and some of the propositions seem reasonable, they are often based on the same biomedical fundamental assumptions, and there is a risk of hijacking the voices of those calling for a real change in order to keep the current biomedical model intact. Programs like these, which only add psychological or social interventions on top of standard care, often with the main goal of increasing compliance to treatment, seem to be failing [130] as the problems are much more fundamental than that. More funds only, more of the same only approach will not solve the current crisis. In fact, there is even research suggesting that an alternative system such as the one proposed here could be cheaper [103,131,132], or at least provide better care with the same funding, especially if we account for reduced spending on drugs, reduced expensive hospital stays, and possibly better social and work functioning of patients long-term. Working on partial solutions that are gradually implemented in the current system is associated with the risk of watering down and co-opting such ideas, which is why conceptualizing a comprehensive alternative and advocating for a systemic change may be a better strategy for a real change.

Recently, important books and papers presenting arguments and solutions partly similar to those presented here were published [133,134,135]. Perhaps the real challenge now, besides countering corporate and guild interests [136], is to come up with a systemic alternative that could actually replace the current model of care. Even if the implementation of such a system currently seems unlikely because of the influence of parties interested in maintaining the status quo, that is to a large degree associated with the neoliberal cultural and economic order [137,138,139], here, we have tried to provide a rough outline of some of the approaches that, together, could form a basis of a novel program for research and an alternative model of care. Of course, more work and addressing crucial details are needed, and this proposition could only be considered to be a first step or a call for moving in a similar direction. Perhaps, changes, as the ones described here, will only be possible in future if the training and education of coming generations of mental health professionals and the general public, now largely influenced by pharmaceutical companies [140], includes this kind of perspective. Effecting changes could require action on different levels; alongside the grassroots movement of psychiatric survivors, scientific outlets open to alternative conceptualizations of mental health such as the journal *Psychosis* [141] are needed, as well as “top-down” efforts such as the recently published World Health Organization [141] guideline for organizing community mental health services. Cooperation between service users, clinicians, researchers, universities, non-governmental organizations and other actors and policy makers is crucial. It might be easier to imagine the end of the world than the end of capitalism [142], and possibly it also seems true in the case of the mental health industry, but we hope that this sketch may provide an impulse and contribution towards imagining a more humane and a more effective mental health care system.
